# Interfacial Composition of Surfactant Aggregates in the Presence of Fragrance: A Chemical Trapping Study

**DOI:** 10.3390/molecules27144333

**Published:** 2022-07-06

**Authors:** Jiani Gong, Kaixin Yao, Qihan Sun, Yujia Sun, Lijie Sun, Changyao Liu, Bo Xu, Jiajing Tan, Li Zhao, Baocai Xu

**Affiliations:** 1 Department of Daily Chemical Engineering, Beijing Technology and Business University, No. 11 Fucheng Road, Beijing 100048, China; gongjiani_jennifer@126.com (J.G.); m15093610601@163.com (K.Y.); sunqihan1999@163.com (Q.S.); 13520372492@163.com (Y.S.); slj2605@163.com (L.S.); zhaol@btbu.edu.cn (L.Z.); xubaoc@btbu.edu.cn (B.X.); 2 McIntire School of Commerce, University of Virginia, Charlottesville, VA 22903, USA; 3 Department of Organic Chemistry, College of Chemistry, Beijing University of Chemical Technology, Beijing 100029, China; tanjj@mail.buct.edu.cn

**Keywords:** surfactants, fragrances, chemical trapping, aggregates, interface, aromatic alcohols

## Abstract

In recent years, there has been increasing interest in daily-use chemical products providing a pleasant scent. The added fragrance molecules may induce microstructural transitions of surfactant aggregates, which further affect the physical and chemical properties of the products. Here, the effects of four types of aromatic alcohols (cinnamyl alcohol, phenyl ethanol, phenyl methanol and anisyl alcohol) on cetyltrimethylammonium bromide (CTAB)/KBr aggregates were studied. The combined results from rheology, dynamic light scattering, and transmission electron microscopy measurements showed that cinnamyl alcohol induced significant micellar growth, while increases in micellar growth were less obvious for the other aromatic alcohols. The changes in the interfacial molarities of water, aromatic alcohol, and bromide ions during such transitions were studied using the chemical trapping method. Transitions resulting from added cinnamyl alcohol were accompanied by significant declines in interfacial water and bromide ion molarities, and a rise in interfacial alcohol molarity. The marked decrease in interfacial water molarity was not observed in previous studies of the octanol induced formation of wormlike micelles and vesicles, indicating that a different mechanism was presented in the current system. Nuclear magnetic resonance investigation showed that π–π stacking between cinnamyl alcohols, but not cation–π interactions between alcohols and CTAB headgroups, facilitated the tight packing of alcohol molecules in CTAB aggregates and the repulsion of water from the interfacial region. The current study may provide a theoretical basis for the morphological regulation of surfactant aggregates in the presence of additives.

## 1. Introduction

Surfactants are amphiphilic molecules that self-assemble spontaneously into aggregates of various types, e.g., spherical and wormlike micelles, uni-lamellar and multi-lamellar vesicles, liquid crystals and so forth [1]. Surfactants have been widely applied to various industrial and consumer products, such as household cleaning agents, personal care and cosmetic products, drug carriers, and oil wet treatment fluids [2,3,4]. The morphologies of surfactant aggregates strongly influence the physical and chemical properties of these products. For instance, wormlike micelles [5], which are usually found in personal care and other products, form gel-like structures and show remarkable rheological properties [3,6]. These giant micelles are also called “living polymers”, which are different from covalently bonded polymers because the giant micelle systems are in a dynamic equilibrium where the “polymers” constantly break down and recombine [7,8]. Moreover, vesicles that encapsulate both hydrophobic and hydrophilic molecules can serve as effective delivery systems [4,9]. Therefore, the precise regulation of surfactant aggregate morphology has always been of great research interest. The driving force of such self-assembly is the hydrophobic effect of the hydrophobic tails of the surfactant molecules. However, the balance of forces that controls the morphologies of the aggregates depends on the delicate interactions among the surfactant’s hydrophilic headgroups, counterions, water, and other additives in the interfacial region between the aggregates and bulk water [10,11].

Nowadays, as living standards improve, there is an increasing demand for products bearing pleasant scents. For instance, scent-releasing fabric softeners can be formulated using cationic vesicles that can deliver fragrance while serving as friction-reducing agents. The fragrance-releasing efficiency is therefore strongly impacted by the interaction between the vesicles and fragrances [9]. Salts and small organic molecules incorporate themselves into aggregates composed of amphiphiles, which alters their microstructures [11,12,13,14,15,16]. The fragrances themselves are also small organic molecules that may induce the microstructural transition of surfactant aggregates. Fieber and coworkers have studied the effect of several fragrance molecules on the formation of wormlike micelles in aqueous solutions of sodium laureth sulfate (SLES) [17,18]. The Ogura group showed that fragrances can increase the bilayer thickness of cationic vesicles composed of TEQ surfactants [9].

Aryl alkyl alcohols are a group of fragrance molecules in which an alkyl alcohol is covalently bonded to a substituted or unsubstituted benzene ring [19]. These aromatic alcohols are widely used as additives in the cosmetic and food industries. Cinnamyl alcohol, phenyl ethanol, and anisyl alcohol, which have hyacinth, rose, and hawthorn-like odours, respectively, are used abundantly in personal care and cosmetic products [20]. Phenyl methanol (benzyl alcohol), which also has a floral odour, is employed as a preservative and cosolvent in personal care and pharmaceutical injection formulations [21]. Studies also show that phenyl ethanol (together with tryptophol and tyrosol) is produced during alcoholic fermentation by yeasts and positively contributes to the flavor of wines [20]. Therefore, the interactions between these aromatic alcohols and surfactants are very important in various fields. It is well known that aromatic molecules promote micellar growth. Aromatic compounds exert important influences on surfactants, such as via phenyl-headgroup interactions [22], hydrophobic/hydrophilic interactions [17], intermolecular hydrogen bonds [23,24] and steric effects [25]. Multiple studies have also shown that alcohols have a significant effect on the physicochemical properties of surfactant solutions [21,26,27,28]. The effect of aliphatic alcohols on surfactant aggregates depends strongly on their chain length. Short-chain alcohols such as methanol and ethanol usually serve as a cosolvent and tend to break down aggregates [29]. However, long-chain alcohols, which act as a kind of cosurfactant, tend to decrease the critical micelle concentration (cmc) of the surfactant and induce micelle growth [12,30,31,32,33,34,35].

Previously, our group [12] employed the chemical trapping method to probe the interfacial composition changes during an octanol induced CTAB/KBr micelle-to-vesicle transition. We showed that the transition is accompanied by a noticeable rise in interfacial water and alcohol molarities, and a decline in interfacial counterion molarity. Such interfacial composition changes were almost absent when butanol was added, and butanol did not trigger obvious microstructural transitions in the same system. This result suggested that the interactions between alcohol, surfactant, and water at the aggregate-water interface played a crucial role in controlling the morphologies of the aggregates. Herein, to better understand the interfacial surfactant-fragrance interactions, we studied the effect of several aromatic alcohols (cinnamyl alcohol, phenyl ethanol, phenyl methanol, and anisyl alcohol), which are also fragrance ingredients, on CTAB/KBr micellar solutions. This was followed by analysis of the interfacial molarity changes in water, alcohols, and counterions during aggregate transitions using the chemical trapping method. This research may provide a theoretical basis for the application of surfactant-fragrance formulations in many fields.

## 2. Materials and Methods

### 2.1. Materials

Cetyltrimethylammonium bromide (CTAB, AR, Beijing Aoboxing Biotech Co., Ltd. Beijing, China) and KBr (≥99%, Macklin Biochemical Co., Ltd., Shanghai, China) were used as received. The cinnamyl alcohol (≥98%), phenyl ethanol (≥99.5%), phenyl methanol (≥99%), and anisyl alcohol (≥98%) were purchased from Shanghai Macklin Biochemical Co., Ltd, Shanghai, China. And used as received. The probe, 16-ArN_2_^+^, and typical dediazoniation products, i.e., 16-ArOH and 16-ArBr, had been prepared previously in the laboratory. The dediazoniation products of the probe and alcohols were obtained from Wuxi AppTec, Shanghai, China, for calibration curve preparation. All aqueous solutions were prepared using deionized water.

### 2.2. Chemical Trapping with a Probe in Aqueous CTAB/KBr/Aromatic Alcohol Solutions

The aqueous solutions of 0.1 M CTAB in the presence 0.05 M KBr were prepared in deionized water and equilibrated for one day. Aromatic alcohols of calculated volume were added to the above mixed solutions for the preparation of 0.1 M CTAB/0.05 M KBr/aromatic alcohol systems. The volume of added aromatic alcohols was extremely small (less than 3%) so that the concentrations of CTAB and KBr remained essentially the same. The samples were kept in a water bath at 25 °C for one day before testing. The chemical trapping reaction was initiated by adding 20 μL freshly prepared stock solutions of 16-ArN_2_BF_4_ dissolved in ice-cold MeCN to 2 mL of the aqueous solutions containing 0.1 M CTAB, 0.05 M KBr, 1 mM HBr, and aromatic alcohols of the needed concentrations. Final probe concentrations were in the range of 1.2 × 10^−5^ ~ 2 × 10^−4^ M. The volumetric flasks were sealed and thermostatted at 25 °C for 48 h. Each sample was diluted with methanol before injecting into HPLC. HPLC measurements were performed on an Agilent Technologies 1200 series equipped with a UV/Vis detector, a ZORBAX Eclipse Plus C18 column and Agilent Chemstation software. The temperature of the chemical trapping experiments was maintained at 25 °C using a water bath (±0.5 °C). Percentage yields were obtained from average values of peak areas from triplicate injections with the appropriate calibration curves.

### 2.3. Rheological Measurements

A Haake MARS III rheometer equipped with a coaxial cylinder sensor system CC25 DIN TI was used in this experiment to perform steady and dynamic rheological measurements. The viscosities of the samples were obtained from steady-shear measurements under a gradient of shear rates from 0.03 to 500 s^−1^. Frequency sweep measurements were performed at a given stress level under a continuously changing frequency ranging from 1 to 100 s^−1^.

### 2.4. Nuclear Magnetic Resonance (NMR) Spectroscopy

^1^H NMR experiments were performed on a Bruker−600 (600 MHz) spectrometer. The signals are quoted as δ values in ppm using the residual protonated solvent (D_2_O) signals which are located at δ = 4.79 ppm as an internal standard. 2D NOESY spectra for the samples were performed with Brucker standard pulse sequences. The experimental data were collected over 2048 complex points. A mixing time of 300 ms, a relaxation delay of 2 s, and an acquisition time of 85 ms were used. A total of 16 scans were carried out.

## 3. Rationale of the Chemical Trapping (CT) Method

The interfacial region between aggregates and bulk water is like a “black box” as it is highly difficult to focus on this specific area using general experimental methods. The CT method, established by the Romsted group, has been employed to determine the interfacial molarities of weakly basic nucleophiles, such as halide ion, water, carboxylate groups, amide bonds, and alcohols, in surfactant aggregate solutions, and the details of the method can be found in previous references [10,11,12,36,37].

**Figure 1:**
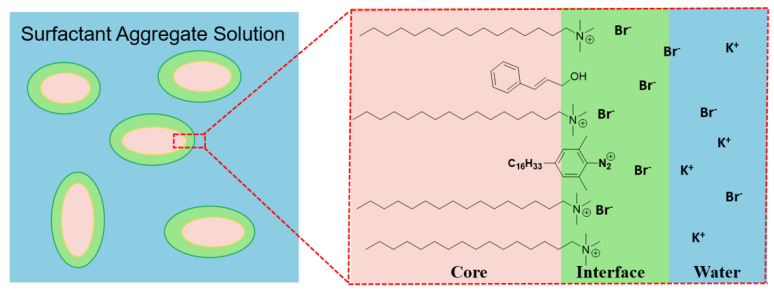
Cartoon showing the location of 16-ArN_2_^+^ at the interface of surfactant aggregates composed of CTAB/KBr/aromatic alcohol, taking cinnamyl alcohol as an example.

**Scheme 1:**
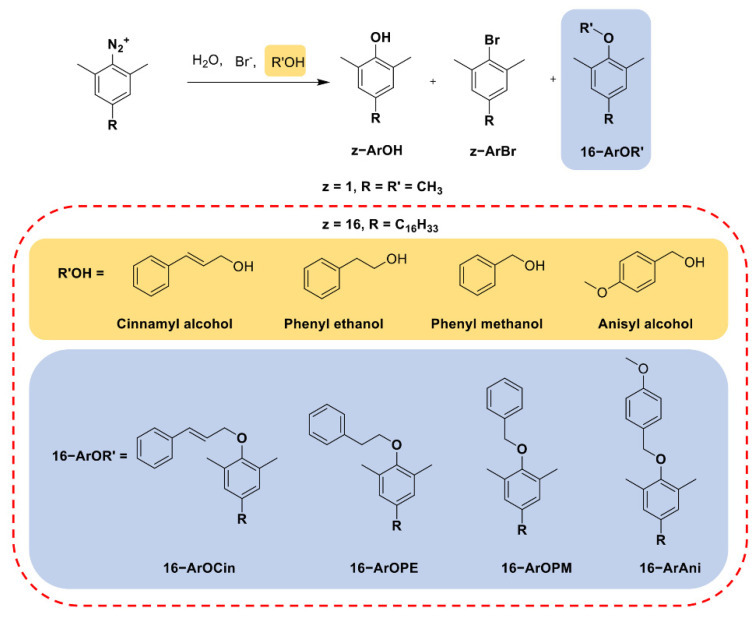
The CT reaction products from the probe in competitive reactions between water, bromide ions, cinnamyl alcohol, phenyl ethanol, phenyl methanol, and anisyl alcohol.

Here, the rationale of the CT method was applied to the CTAB/KBr/aromatic alcohol solutions; this method was similar to one we previously described for the CTAB/KBr/long-chain alcohol [12], and is summarized below in brief. The long-chain probe (2,6-dimethyl−4-hexadecylbenzenediazonium ion, 16-ArN_2_^+^) was added to the CTAB/KBr/aromatic alcohol aggregate solutions. The 16-ArN_2_^+^ molecules locate themselves in the interfacial region where they react, as shown in Figure 1 (taking cinnamyl alcohol as an example). The CT reaction pathways with the probe in CTAB/KBr/aromatic alcohol aqueous solutions are summarized in Scheme 1. The selectivities of the probe towards these nucleophiles (water, bromide ions, and alcohols) are determined by similar reactions that occur with a short-chain probe (2,4,6-trimethylbenzenediazonium ion or 1-ArN_2_^+^) in reference solutions [12]. Therefore, the interfacial molarities of water, H_2_O_m_, bromide ions, Br_m_, and aromatic alcohols, ROH_m_, were determined along with their corresponding product yields %16-ArOH, %16-ArBr, and %16-ArOR’, respectively, through the selectivity determined by a short-chain probe. The dediazoniation products corresponded to cinnamyl alcohol (CinOH), phenyl ethanol (PEOH), phenyl methanol (PMOH), and anisyl alcohol (AniOH), which are 16-ArOCin, 16-ArOPE, 16-ArOME, and 16-ArOAni, respectively.

## 4. Results and Discussion

### 4.1. Aromatic Alcohols Induced Morphological Transitions of the CTAB/KBr System

**Figure 2:**
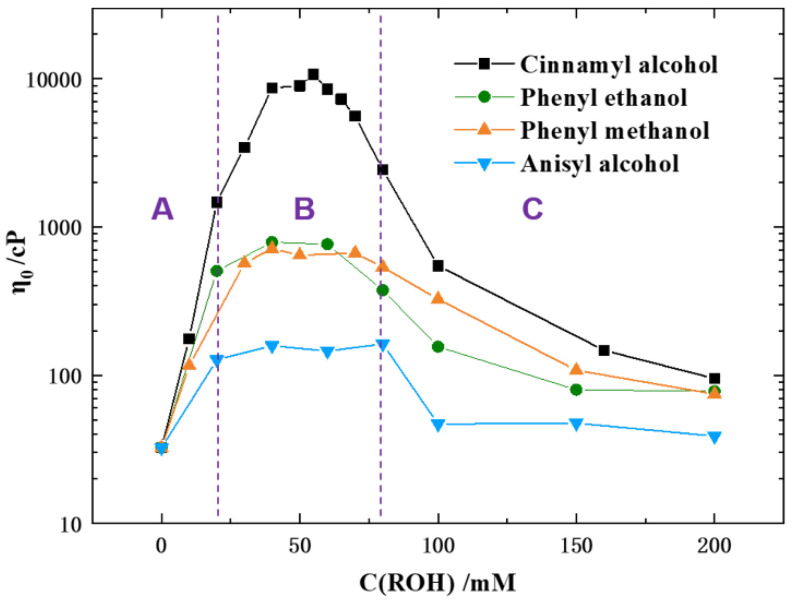
Effect of cinnamyl alcohol (CinOH), phenyl ethanol (PEOH), phenyl methanol (PMOH), and anisyl alcohol (AniOH) concentrations on the *η_0_* value of 0.1 M CTAB/0.05 M KBr solutions. The purple dotted lines divide the graphs into three regions (A, B and C).

Although the morphological transitions in similar CTAB/salt/alcohol systems have been reported many times [32,33], the morphological transitions of the current CTAB/KBr/fragrance systems were studied briefly to provide a point of comparison for the chemical trapping results. Figure 2 shows the effects of different concentrations of a class of aromatic alcohols on the zero-shear viscosity (*η_0_*) of aqueous surfactant solutions containing 0.1 M CTAB, 0.05 M KBr and 0–200 mM alcohols at 25 °C. Four types of aromatic alcohols, namely, cinnamyl alcohol (CinOH), phenyl ethanol (PEOH), phenyl methanol (PMOH), and anisyl alcohol (AniOH), were studied. The corresponding steady shear viscosities of these mixed solutions versus shear rate are shown in Figure S1 in Supplemental Information. Figure 2 shows that the *η_0_* value depends strongly on the type and concentration of the aromatic alcohol. For all types of aromatic alcohol, the *η_0_* values increased with increasing ROH concentration (Region A) to a maximum (Region B), followed by a decrease as ROH concentrations further increased to a higher concentration range (Region C). The maximum *η_0_* values for different alcohol systems were achieved at a roughly similar concentration range; however, the absolute *η_0_* values were significantly different for different alcohol types and were in the order of CinOH > PEOH ~ PMOH >AniOH. For cinnamyl alcohol, the maximum *η_0_* value reached ca. 10^4^ cP, which was much higher than for the other three types of alcohols, in the alcohol concentration range of 40–65 mM. This change in viscosity in the CTAB/KBr/CinOH system was noteworthy, at over three orders of magnitude, and was associated with the formation of wormlike micelles [12,33]. However, compared to CinOH, the *η_0_* value changes for the other three types of alcohols were relatively small, and the maximum *η_0_* values were around 700~800 cP for PEOH and PMOH, and 100~200 cP for AniOH. Previously, we studied the effect of adding octanol and butanol on the rheological properties of a 0.1 M CTAB/0.1 M KBr system, and showed that octanol triggers the formation of wormlike micelles whereas butanol did not [12]. The maximum *η_0_* value for the octanol system was about 10^4^ cP, which is similar to that of the 0.1 M CTAB/0.05 KBr/CinOH system, although the salt concentration of the CinOH system was only half of that of the octanol system. Furthermore, compared to butanol which hardly changed the *η_0_* value at all, PEOH, PMOH, and AniOH did increase the *η_0_* value to some extent.

**Figure 3:**
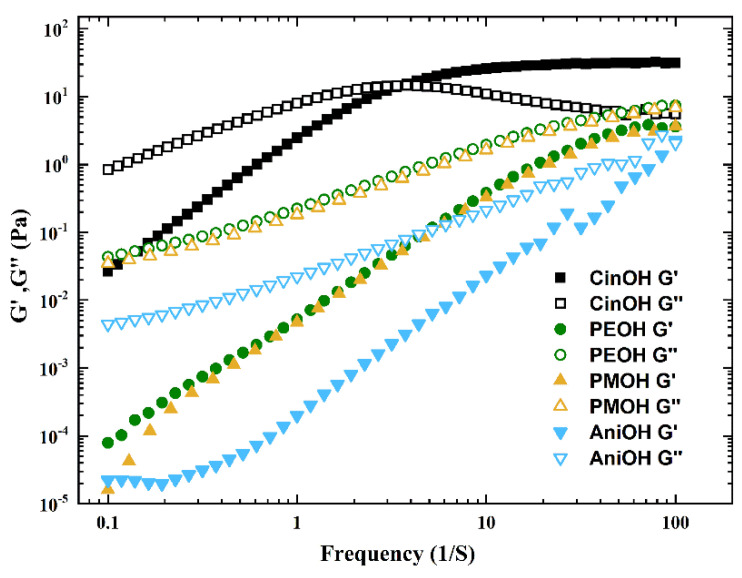
Changes in G’ (■● 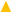

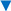
) and G’’ (□
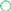

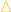

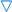
) as a function of oscillation frequency (*ω*) for the solutions of CTAB/KBr in the presence of 55 mM CinOH (black squares, ■□), 40 mM PEOH (oval circles, ●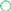), 40 mM PEOH (orange triangles, 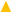

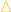
) or 40 mM AniOH (blue triangles
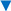

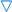
) at 25 °C.

The results in Figure 2, with added CinOH, indicate the presence of long wormlike micelles that gave rise to a strong network. For PEOH, PMOH, and AniOH, there were also rodlike or wormlike micelles which were much shorter. To further confirm this, dynamic rheological measurements were employed. In angular frequency sweep tests, the variations in elastic modulus (G’) and viscous modulus (G’’) as a function of oscillation frequency, *ω*, at 25 °C for the solutions of CTAB/KBr/Aromatic alcohol were studied and are shown in Figure 3. The concentrations of aromatic alcohols were selected from the concentrations corresponding to the highest *η_0_* values in Figure 1; these were 55 mM for CinOH, and 40 mM for PEOH, PMOH, and AniOH. Figure 3 illustrates that the viscoelastic behaviours of the CinOH system were different from those of the other three aromatic alcohols. For PEOH, PMOH, and AniOH, the G’’ value (open symbols) was always higher than the G’ value (closed symbols), although these manifested as viscoelastic behaviours. However, for CinOH, there was a point of intersection between the G’ line and G’’ line, with a larger elastic modulus at high frequencies, but a larger viscous modulus at low frequencies. Such Maxwell fluid-like behaviour is typical for wormlike micelles [38,39]. Therefore, both steady and dynamic rheological measurements confirmed the formation of giant micelles in the presence of CinOH.

The aggregation behaviours of CTAB/KBr in the presence of aromatic alcohols were also studied by dynamic light scattering (DLS) and transmission electron microscopy (TEM), the results of which are shown in the Supplementary Information. Figure S2 shows that, in all systems, there were always large peaks at ca. 10 nm and smaller peaks at 100–200 nm; these peaks coexisted when the alcohol concentration was equal to or below 100 mM. The main peak was at ~10 nm, indicating that the micelles were not simply spherical micelles, which should be around 1–2 nm, but ellipsoidal or rodlike micelles. The peaks at around 100 nm showed the coexistence of vesicles, which was further confirmed by the TEM photos (Figure S3). This result is also consistent with a previous study [12]. The DLS results also showed that the fraction of vesicles increased at 200 mM CinOH, but almost disappeared in the presence of 200 mM PEOH, PMOH, and AniOH.

### 4.2. Changes in Interfacial Compositions Probed by Chemical Trapping

The CT method was employed to estimate the changes in interfacial water, bromide ion, and aromatic alcohol molarities of CTAB/KBr/ROH systems with increasing stoichiometric concentrations of added alcohols. The probe 16-ArN_2_^+^ was added into solutions of CTAB/KBr containing 0–200 mM aromatic alcohols (CinOH, PEOH, PMOH, and AniOH) in the presence of 1 mM HBr at 25 °C. The weakly basic nucleophiles in these solutions, i.e., water, bromide ion, CinOH, PEOH, PMOH, and AniOH, reacted competitively with the probe in the interfacial region and produced dediazoniation products of 16-ArOH, 16-ArBr, 16-ArOCin, 16-ArOPE, 16-ArOPM, and 16-ArOAni, respectively. These products were analyzed by HPLC, and the corresponding results, including peak areas and observed and normalised yields for CinOH, PEOH, PMOH, and AniOH, are listed in Table 1 and Tables S1–S3 (SI), respectively. The peak areas, which were the average values of triplicate injections, were converted into observed yields using calibration curves. The total observed yield was not 100% because of experimental errors and the formation of by-products. However, the ratios between %16-ArOH, %16-ArBr, and %16-ArOR’ are most important, rather than their absolute values. Therefore, these observed yields were further normalized.

**Figure 4:**
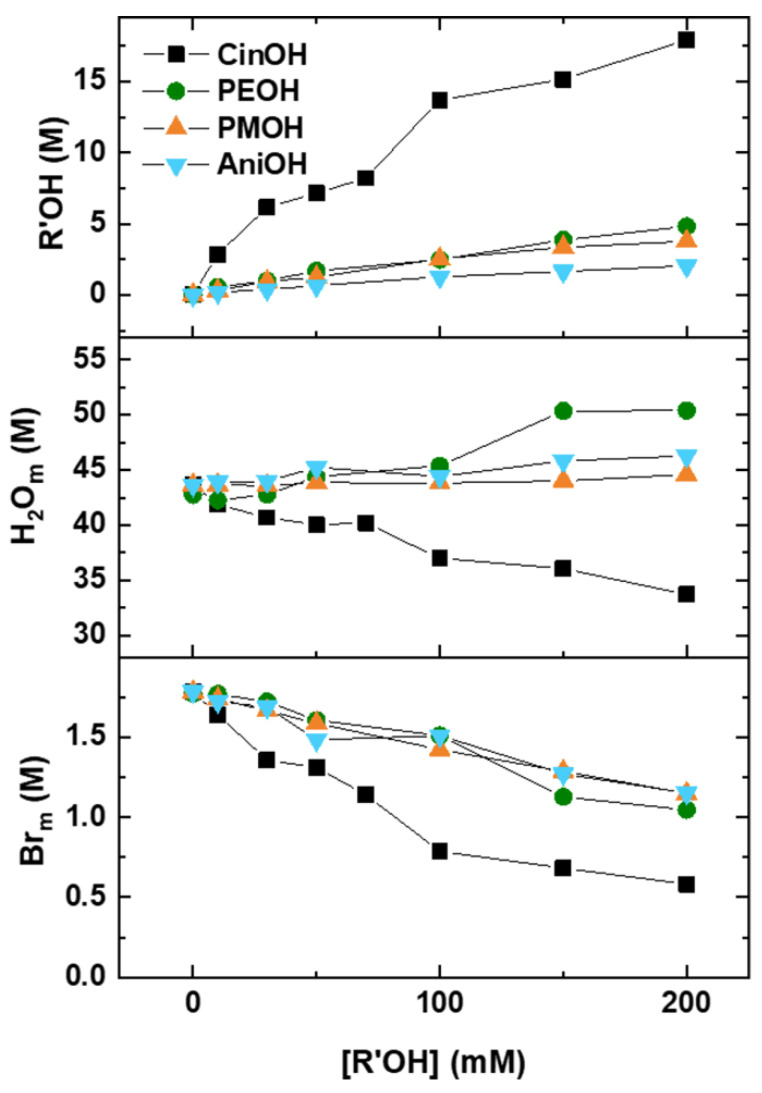
Interfacial molarities of alcohol, water, and counterions in surfactant aggregates with increasing stoichiometric concentrations of added CinOH (black squares), PEOH (green circles), PMOH (orange triangles), and AniOH (blue triangles).

Figure 4 shows the interfacial molarities of alcohols (CinOH_m_, PEOH_m_, PMOH_m_, AniOH_m_), water (H_2_O_m_), and bromide counterions (Br_m_) in CTAB/KBr aggregates versus the stoichiometric concentration of added alcohols. The calculations were the same as those for CTAB/KBr/long-chain alcohol systems [12] and are summarised in the footnotes of Tables S4–S7 (SI). As the stoichiometric concentration of alcohol was increased, the interfacial molarities of alcohol increased and those of bromide ions decreased. The interfacial water molarity decreased strongly with increasing [CinOH] but remained almost the same for other alcohols. When CinOH was added at 0.2 M, the interfacial water molarity decreased from ca. 43 M to ca. 33 M, and the interfacial counterion molarity decreased from ca. 1.7 M to ca. 0.6 M. However, the interfacial water and counterion molarities were in the range of 44–47 M, and 1.0–1.2 M, which are lower compared to the other aromatic alcohols at 0.2 M. Thus, the interfacial water and bromide ion molarities of the CinOH system were much lower than those of other aromatic alcohol systems. Such significant decreases in the water and counterion molarities for CinOH were related to the marked increase in interfacial alcohol molarity. With the addition of 0.2 M aromatic alcohol, the interfacial molarities of CinOH, PEOH, PMOH, and AniOH increased from 0 to 17.9 M, 4.3 M, 3.8 M, and 2.1 M, respectively. At a fixed alcohol concentration, the interfacial alcohol molarities followed the trend: CinOH_m_ > PEOH_m_ ~ PMOH_m_ > AniOH_m_. This order was consistent with the trend of maximum zero-shear viscosity in Figure 2, indicating that the CT method may have potential for maximum zero-shear viscosity prediction. Moreover, although the AniOH_m_ value is the lowest in the studied four systems, it is still ten times higher than the stoichiometric concentration of added alcohol (0.2 M), indicating that the aromatic alcohols were mainly concentrated at the interfacial region of aggregates.

The results in Figure 4 also indicate that the interfacial molarity changes were strongly correlated with the morphologies of surfactant aggregates. We have shown that, among the four types of aromatic alcohols, CinOH triggered the strongest micellar growth. Interestingly, the CT results also showed that the interfacial composition of the CinOH system changed much more significantly than the other alcohol systems. The formation of giant micelles is accompanied by a marked increase in interfacial CinOH molarity and a decrease in the interfacial water and bromide ion molarities. POH, PMOH, and AniOH produced similar but moderate interfacial molarity changes for the alcohol and bromide ions, with the concentration of added alcohol associated with the impact of maximum zero-shear viscosity to some extent.

The increase in interfacial CinOH molarity can be divided into two stages. The first stage almost levelled off at the same stochiometric CinOH concentration when the maximum zero-shear viscosity was obtained, indicating that the micellar growth was accompanied by a strong increase in interfacial alcohol molarity. The second stage was also significant and may be explained by the formation of vesicles in these solutions. Figure S2 (SI) shows that the peak at around 100 nm increased strongly at 200 mM CinOH, but disappeared with the addition of other alcohols at the same concentration. Such phenomena were consistent with strong interfacial dehydration at high CinOH stochiometric concentrations.

### 4.3. Mechanism for the Tight Packing of CinOH in CTAB Aggregates by Nuclear Magnetic Resonance (NMR)

Compared to linear alcohols, aromatic alcohols have a much stronger tendency to enter into aggregates. Previously we have shown that the interfacial octanol molarity was 0.9 M in 0.1 M CTAB/0.1 M KBr solutions containing 55 mM octanol. Here, at an added aromatic alcohol concentration of 50 mM, the interfacial molarities of CinOH_m_, PEOH_m_, PMOH_m_, and AniOH_m_ were 7.2 M, 1.7 M, 1.3 M, and 0.7 M, respectively. Although the number of carbons on these alcohols is quite comparable with octanol (except for AniOH), the interfacial alcohol molarity values of aromatic alcohols were higher than that of octanol.

We hypothesised that possible interactions such as π–π stacking [40] between aromatic alcohols and cation- p interactions [41,42] between the headgroups and aromatic alcohols may facilitate the tight packing of alcohol molecules in surfactant aggregates. Such interactions were characterised by NMR techniques, which have been successfully utilised for identifying cation-π or π-π interactions in aromatic solubilised species within surfactant micelles [40,41,42].

**Figure 5:**
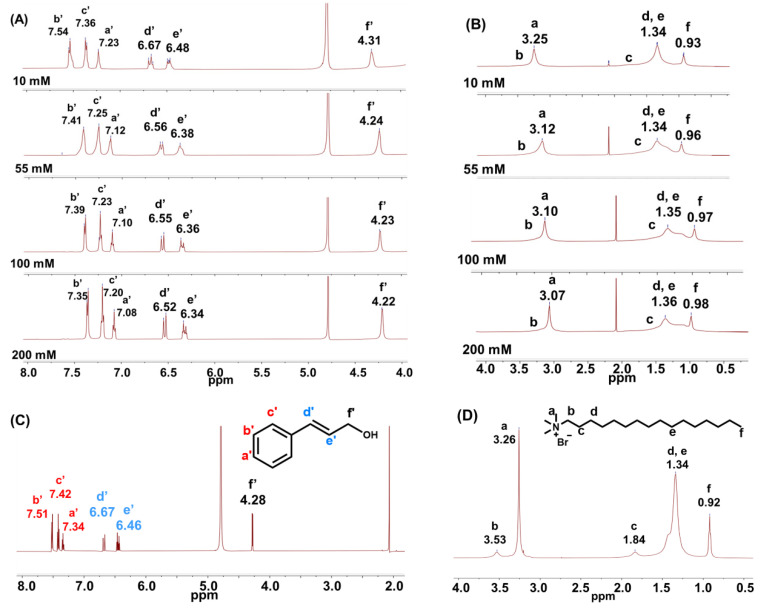
^1^H NMR spectra of (**A**) CinOH and (**B**) CTAB in 0.1 M CTAB/0.05 M KBr/CinOH aqueous solutions at different alcohol concentrations in D_2_O; (**C**) ^1^H NMR spectra of 55 mM CinOH (in the absence of aggregates) and (**D**) 0.1 M CTAB/0.05 M KBr (in the absence of CinOH) in D_2_O.

The mutual orientation of solubilised aromatic alcohols with CTAB aggregates was studied using ^1^H NMR spectroscopy. Figure 5A,B show the ^1^H NMR spectra of CinOH and CTAB in 0.1 M CTAB/0.05 M KBr/CinOH aqueous solutions at different alcohol concentrations in D_2_O. As the control group, Figure 5C,D show the corresponding chemical shifts of 55 mM CinOH (without added CTAB and KBr) and 0.1 M CTAB/0.05 M KBr solution (without added CinOH) in D_2_O, respectively. In the absence of surfactant aggregates, the protons on the CinOH benzene ring resonate at 7.34–7.51 ppm, and the protons on the side chain resonate separately at 6.67 ppm and 6.46 ppm. However, in the presence of CTAB, the protons of the 55 mM CinOH benzene ring appeared considerably upfield at 7.12–7.41 ppm, and the side chain protons also appeared upfield at 6.56 ppm and 6.38 ppm. Such an overall upfield shift of the CinOH protons indicated that the environment of those protons became relative nonpolar, and may imply π–π stacking of the CinOH alcohols in the CTAB aggregates [43]. With increasing CinOH concentration, Figure 5A also shows a gradual overall upfield shifting of CinOH protons, which indicates increasing π–π stacking interactions between CinOH molecules. The gradual upfield shifting of the N-Me protons with increasing CinOH concentration indicates an increase in molecular packing in the interfacial region of the aggregates, as shown in Figure 5B,D. Such evidence also overturns our previous hypothesis regarding the strong cation- p interactions between the CinOH and CTAB headgroup. Previous studies [41,42] have shown that such cation- p interactions would bring aromatic molecules to a more polar interfacial region and induce downfield shifting of the protons on the aromatic rings. The ^1^H NMR spectra of AniOH in 0.1 M CTAB/0.05 M KBr/AniOH aqueous solutions at different alcohol concentrations in D_2_O are also summarised in Figure S4 (SI). Similar results were observed, but the signals shifted less. These results imply that the π-π stacking interaction is much stronger for CinOH than for AniOH.

**Figure 6:**
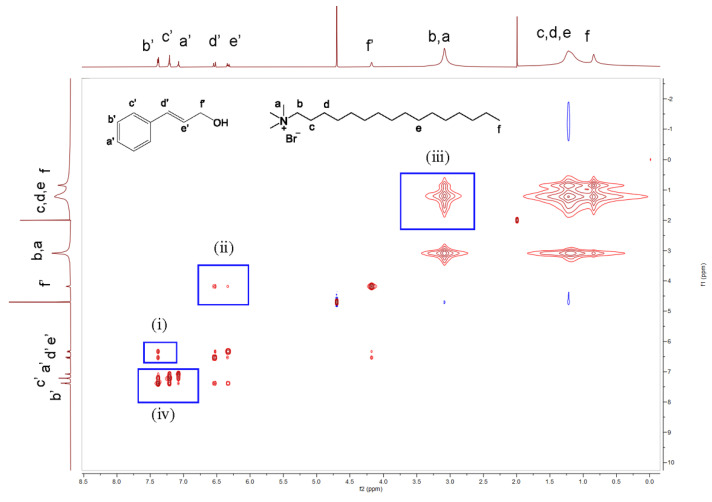
2D NOESY spectrum of 0.1 M CTAB/0.05 M KBr/55 mM CinOH in D_2_O.

Nuclear Overhauser effect spectroscopy (NOESY) is a 2D NMR technique based on correlation via dipolar interaction between protons that are located closer than 5 Å in space. The NOESY spectra of the 0.1 M CTAB/0.05 M KBr/55 mM CinOH system are recorded in Figure 6. The obvious cross-peaks between any two protons of the b’, c’, and a’ peaks correlate to the benzene ring protons (box iv), whereas cross-peaks were observed between protons b’, c’, and a’ and the N-Me protons of CTAB. This shows that the space between the CinOH benzene rings is close, and there exists a strong π-π effect rather than the cation- π effect that was previously suspected. The π-π effect leads to face-to-face stacking, which was favourable for micelle formation.

Obvious cross-peaks between b’/d’ and e’ (box i), and f’/d’ and e’ (box ii) appeared, suggesting that the space between the side chain protons and benzene ring protons of CinOH is close. Additionally, the double bond of the side chain is conjugated with the benzene ring. At the same time, these data indicate that the side chain protons are located physically close to the ortho protons near the hydroxyl group. Cross-peaks between b, a/c, d, e, and f (box iii), which were observed in the current system, correlate to the N-Me protons of CTAB and the intermediate as well as the terminal methyl protons of the other CTAB. This is considered to have been caused by the interactions at the end-cap region of the rod/wormlike micelles, according to a previous study [41].

A NOESY spectra study of AniOH was also carried out and depicted in Figure S5 (SI). Compared to CinOH, the frequency of occurrence of cross-peaks between benzene ring protons of AniOH (c’’/b’’) was significantly less (Figure S5 box V). There were no cross-peaks between the benzene ring protons c’’ and b’’/f’’, which means that the benzene ring protons were not near the side chain hydroxyl group. According to the electrostatic model proposed by Hunter [44], the electron effect of substituents on benzene rings directly affects π -π stacking, and the electron donor effect is not beneficial to π–π stacking. The methoxy group on the benzene ring of AniOH acts as a conjugated electron donor, which may destroy π–π stacking to some extent.

In summary, the strongest π–π stacking interaction was obtained for CinOH whose double bond on the side chain is conjugated with the benzene ring. This prevents the free rotation of the side chain and gives rise to the highest interfacial molarities of CinOH_m_. However, the methoxy group on the benzene ring of anisyl alcohol may destroy the π–π stacking to some extent, resulting in the lowest interfacial molarities for AniOH_m_. Another piece of evidence to support our hypothesis is that the interfacial water molarity decreased when CinOH was added but increased slightly with added octanol. In a previous paper, the added octanol remained hydrated and increased the interfacial volume; however, for CinOH, the π–π interactions may allow alcohol molecules to pack closely without bringing in water molecules. In fact, these added CinOH molecules in the interfacial region may drive water away from the interfacial region.

## 5. Conclusions

Our current investigations of the CTAB/KBr/aromatic alcohol system showed that the addition of cinnamyl alcohol induced micellar growth. Such growth was less extensive upon addition of other aromatic alcohols, such as phenyl ethanol, phenyl methanol, and anisyl alcohol. CT results showed that such transitions induced by cinnamyl alcohol were accompanied by a significant decrease in interfacial water molarity and an increase in interfacial alcohol molarity. NMR results indicated that the strong π-π stacking interaction between cinnamyl alcohols, but not the cation–π interactions between alcohols and CTAB headgroups, facilitated the tight packing of alcohol molecules. This is also consistent with the noteworthy increase in interfacial CinOH molarity. These tightly packed CinOH molecules enter the aggregates and expel water from the interfacial region, resulting in a noticeable decrease in interfacial water molarity.

## Figures and Tables

**Table 1:** HPLC average peak areas, and observed and normalised (subscript N) product yields for the dediazoniation reaction of 16-ArN_2_^+^ in solutions of 0.1 M CTAB/0.05 M KBr in the presence of 0–200 mM cinnamyl alcohol at 25 °C. [HBr] = 1 mM.

[CinOH]	Peak Areas (10^2^ mAU·s)			Observed Yields (%)				Normalized Yields (%)		
(mM)	16-ArOH	16-ArBr	16-ArOCin	16-ArOH	16-ArBr	16-ArOCin	Total	16-ArOH_N_	16-ArBr_N_	16-ArOCin_N_
0	30.42	23.50	0	69.3	29.4	0	98.7	70.2	29.8	0
10	22.45	17.05	4.19	62.0	25.8	4.23	92.0	67.3	28.1	4.60
30	20.14	14.16	8.88	55.9	22.2	9.00	87.1	64.2	25.6	10.3
50	16.99	11.43	8.30	64.5	23.8	11.6	99.9	64.6	23.9	11.6
70	16.75	10.25	9.34	63.6	21.4	13.0	97.9	64.9	21.8	13.3
100	13.72	7.02	13.98	57.3	16.1	21.4	94.8	60.5	17.0	22.6
150	14.98	7.04	17.23	56.9	14.7	24.0	95.5	59.6	15.4	25.1
200	13.50	6.04	19.61	51.3	12.6	27.3	91.1	56.3	13.8	29.9

## Data Availability

Not applicable.

## Supplementary Materials

The following supporting information can be downloaded at: https://www.mdpi.com/article/10.3390/molecules27144333/s1, Figure S1: The steady shear viscosity as a function of shear rate for the aqueous solutions of 0.1 M CTAB containing 0.05 M KBr and (A) 0–300 mM cinnamyl alcohol, (B) 0–300 mM phenyl ethanol, (C) 0–300 mM phenyl methanol or (D) 0–300 mM anisyl alcohol.; Figure S2: Hydrodiameters of aggregates of 0.1M CTAB/0.05M KBr in the presence of different concentrations of 0–200 mM CinOH (A), PEOH (B), PMOH (C), and AniOH (D) at 25 °C.; Figure S3: TEM micrographs of 0.1 M CTAB/0.05 M KBr in the presence of 55 mM (A) and 70 mM (B) cinnamyl alcohol.; Figure S4. 1H NMR spectra of (A) AniOH & (B) 0.1 M CTAB in 0.1M CTAB/0.05M KBr/AniOH system (C) 80 mM AniOH in D_2_O.; Figure S5. 2D NOESY spectrum of 0.1M CTAB/0.05M KBr/80 mM AniOH in D_2_O.; Table S1: HPLC average peak areas, observed and normalized (subscript N) product yields for dediazoniation reaction of 16-ArN_2_+ in solutions of 0.1 M CTAB/0.05 M KBr in the present of 0–200 mM Phenethyl alcohol at 25 °C. [HBr] = 1 mM.; Table S2: HPLC average peak areas, observed and normalized (subscript N) product yields for dediazoniation reaction of 16-ArN_2_+ in solutions of 0.1 M CTAB/0.05 M KBr in the present of 0–200 mM phenyl methanol at 25 °C. [HBr] = 1 mM.; Table S3: HPLC average peak areas, observed and normalized (subscript N) product yields for dediazoniation reaction of 16-ArN_2_+ in solutions of 0.1 M CTAB/0.05 M KBr in the present of 0–200 mM anisyl alcohol at 25 °C. [HBr] = 1 mM.; Table S4: Estimated values of interfacial molarities of Br_m_, H_2_O_m_, and CinOH_m_, in aqueous solutions of 0.1 M CTAB/0.05 M KBr containing 0–200 mM cinnamyl alcohol at 25 °C. [HBr] = 1 mM.; Table S5: Estimated values of interfacial molarities of Br_m_, H_2_O_m_, and PEOH_m_, in aqueous solutions of 0.1 M CTAB/0.05 M KBr containing 0–200 mM phenylethyl alcohol at 25 °C. [HBr] = 1 mM.; Table S6: Estimated values of interfacial molarities of Br_m_, H_2_O_m_, and PMOH_m_, in aqueous solutions of 0.1 M CTAB/0.05 M KBr containing 0–200 mM phenyl methyl alcohol at 25 °C. [HBr] = 1 mM.; Table S7: Estimated values of interfacial molarities of Br_m_, H_2_O_m_, and AniOH_m_, in aqueous solutions of 0.1 M CTAB/0.05 M KBr containing 0–200 mM anisyl alcohol at 25 °C. [HBr] = 1 mM. Reference [45] is cited in the supplementary materials

## Sample Availability

Samples of the compounds are available from the authors.
