# Supplementary material for: Interfacial Composition of Surfactant Aggregates in the Presence of Fragrance: A Chemical Trapping Study
Source: Molecules. 2022 Jul 6;27(14):4333. doi: 10.3390/molecules27144333 (PMC9320350; doi:10.3390/molecules27144333)
Supplement: Supplementary file 1 [file molecules-27-04333-s001.zip › molecules-1802021-supplementary.pdf]

Supplementary Information

Interfacial composition of surfactant aggregates in the presence of fragrance: a chemical trapping study

Jiani Gong[1], Kaixin Yao[1], Qihan Sun[1], Yujia Sun[1], Lijie Sun[1], Changyao Liu[1]*, Bo Xu[2]*, Jiajing Tan[3], Li Zhao[1], Baocai Xu[1]

1. *Department of Daily Chemical Engineering, Beijing Technology and Business University, No. 11 Fucheng Road, Beijing, 100048, People's Republic of China*
2. *McIntire School of Commerce, University of Virginia, Charlottesville, VA, 22903, U.S.*
3. *Department of Organic Chemistry, College of Chemistry, Beijing University of Chemical Technology, Beijing 100029, P.R. China*

*Corresponding Author.
E-mail: changyaoliu@btbu.edu.cn (C. Liu); bx2pw@virginia.edu (B. Xu)

Table of Contents

Section I: Rheological measurements S2
Section II: Dynamic light scattering (DLS) and transmission electron microscopy (TEM) measurements S2
Section III: HPLC average peak areas, observed and normalized product yields S3
Section IV: Interfacial molarities S7
Section V: NMR results for AniOH S9

Section Ⅰ: Rheological measurements

Figure S1. The steady shear viscosity as a function of shear rate for the aqueous solutions of 0.1 M CTAB containing 0.05 M KBr and (A) 0 – 300 mM cinnamyl alcohol, (B) 0 – 300 mM phenyl ethanol, (C) 0 – 300 mM phenyl methanol or (D) 0 – 300 mM anisyl alcohol.

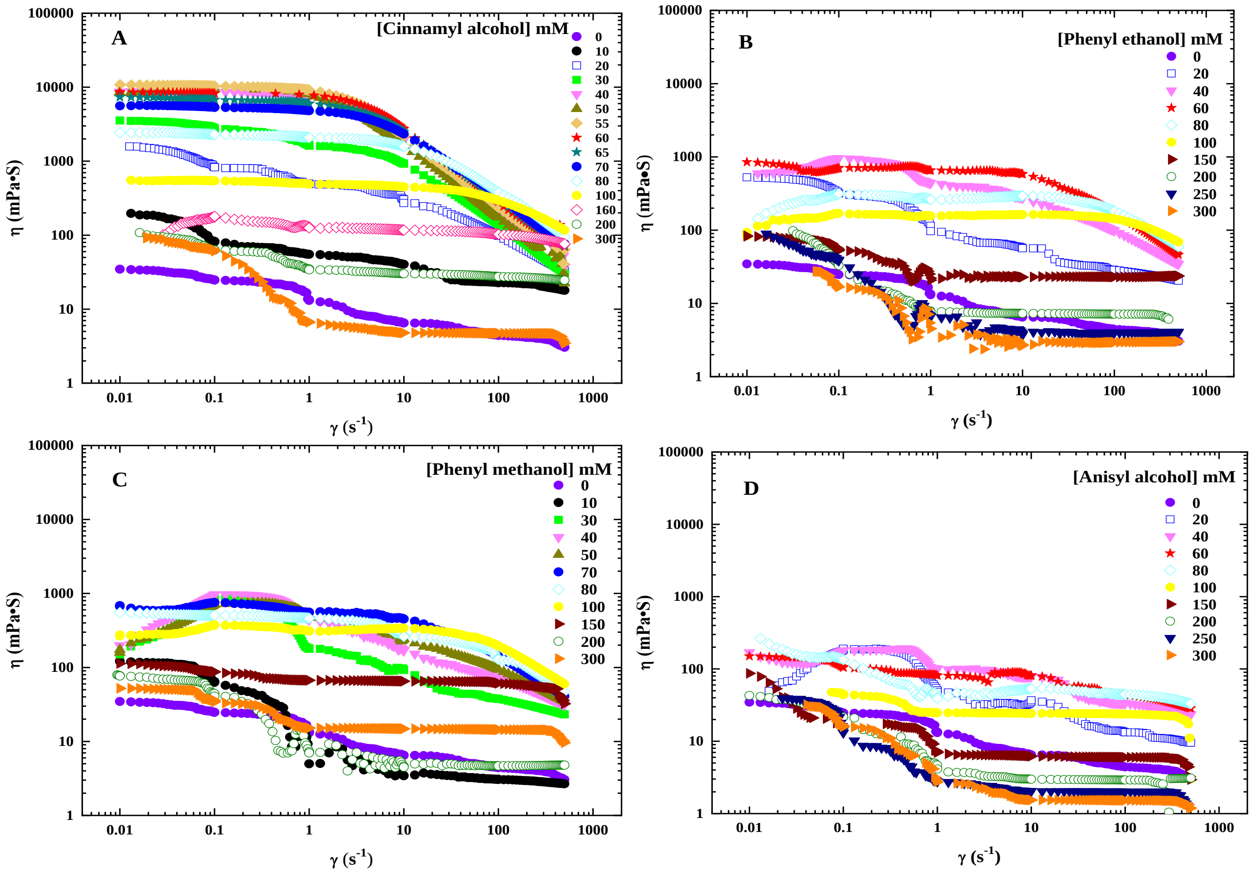

Section II: Dynamic light scattering (DLS) and transmission electron microscopy (TEM) measurements

The hydrodiameters of the surfactant aggregates of 0.1M CTAB/0.05M KBr in the presence of 0 – 200 mM CinOH, PEOH, PMOH and AniOH, respectively, were studied by DLS, **Figure S2**. Measurements of size in diameter for samples were carried out at 25ºC by using a dynamic light scattering instrument (Malvern Zetasizer Nano ZS) equipped with a He-Ne laser. The wavelength was 632.8nm.

Figure S2. Hydrodiameters of aggregates of 0.1M CTAB/0.05M KBr in the presence of different concentrations of 0 – 200 mM CinOH (A), PEOH (B), PMOH (C), and AniOH (D) at 25°C.

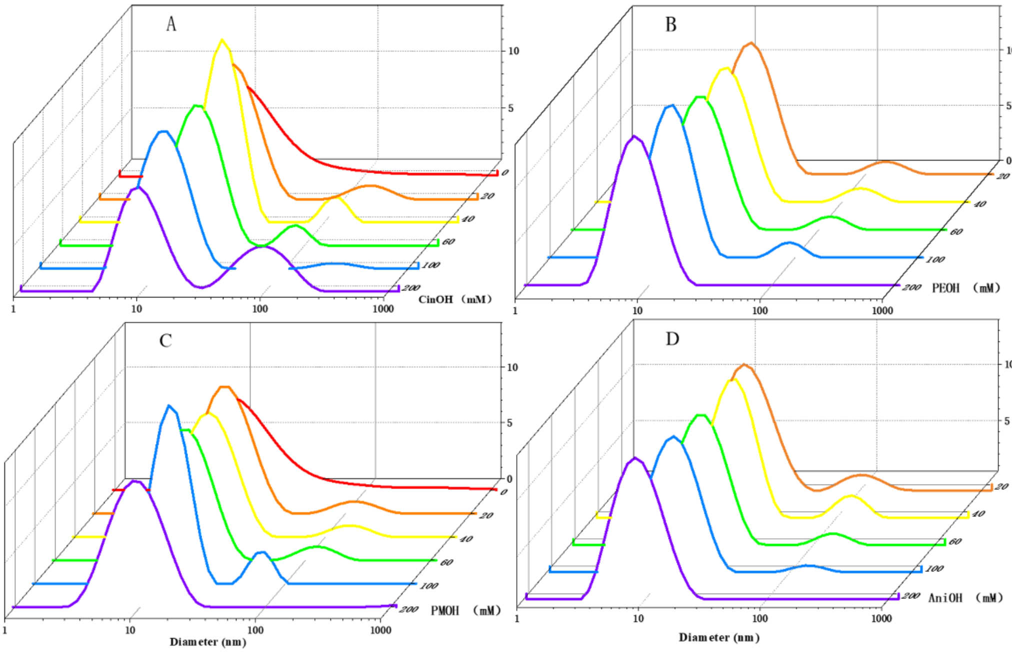

Figure S3. TEM micrographs of 0.1 M CTAB/0.05 M KBr in the presence of 55 mM (A) and 70 mM (B) cinnamyl alcohol

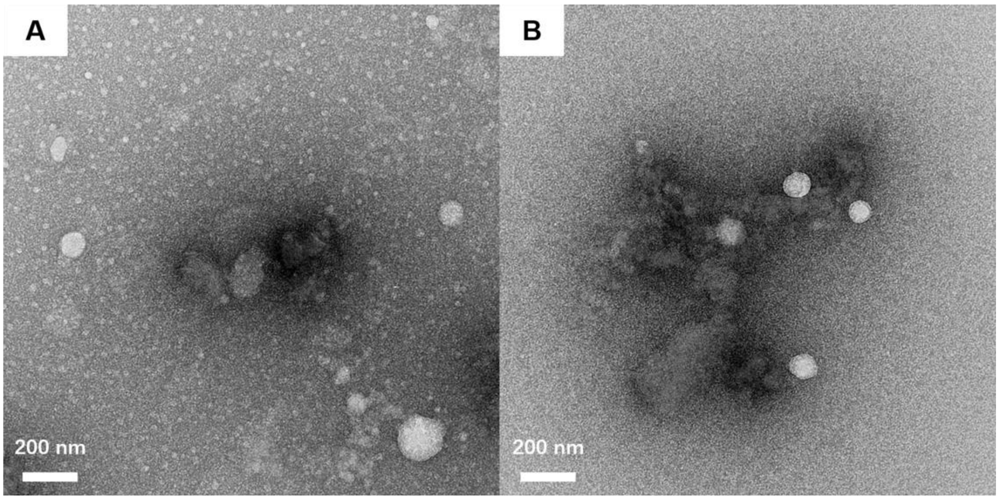

Section III HPLC average peak areas, observed and normalized product yields

The conditions for chemical trapping reaction and HPLC analysis are summarized here. The reaction time was around 48 hours. Prior to HPLC analysis, the product mixture was mixed with an equal volume of methanol. The concentrations of 16-ArN_2^+ were $1.2 \times 10^{-5} – 2 \times 10^{-4}$ M. 100 μL sample injections. Eluting solvents: 65%MeOH/35%*i*-PrOH; Flow rate: 0.4 mL/min; Detector wavelength: 220 nm.

Table S1 – S3 summarized the chemical trapping results, including HPLC average peak areas, observed and normalized product yields, for PEOH, PMOH and AniOH, respectively.

Table S1. HPLC average peak areas, observed and normalized (subscript N) product yields for dediazoniation reaction of 16-ArN_2^+ in solutions of 0.1 M CTAB/0.05 M KBr in the present of 0 – 200 mM Phenethyl alcohol at 25ºC. [HBr] = 1 mM.

[PEOH]	Peak Areas (10^2 mAU·s)			Observed Yields (%)				Normalized Yields (%)		
(mM)	16-ArOH	16-ArBr	16-ArOPE	16-ArOH	16-ArBr	16-ArOPE	Total	16-$ArOH_N$	16-$ArBr_N$	16-$ArOPE_N$
0	30.42	23.50	0	69.3	29.4	0	98.7	70.2	29.8	0
10	31.29	24.33	0.76	67.9	29.0	0.91	97.8	69.5	29.7	0.93
30	29.65	22.40	1.26	64.3	26.7	1.51	92.5	69.6	28.9	1.63
50	29.61	14.67	1.67	64.2	17.5	1.99	83.7	76.9	20.8	2.37
100	27.14	18.33	2.71	58.9	21.8	3.22	83.9	70.2	26.0	3.84
150	23.86	12.33	3.29	72.5	20.6	5.48	98.5	73.6	20.9	5.57
200	24.83	12.06	4.33	75.4	20.1	7.22	102.7	73.4	19.6	7.03

Table S2. HPLC average peak areas, observed and normalized (subscript N) product yields for dediazoniation reaction of 16-ArN_2^+ in solutions of 0.1 M CTAB/0.05 M KBr in the present of 0 – 200 mM phenyl methanol at 25ºC. [HBr] = 1 mM.

[PMOH]	Peak Areas (10^2 mAU·s)			Observed Yields (%)				Normalized Yields (%)		
(mM)	16-ArOH	16-ArBr	16-ArOPM	16-ArOH	16-ArBr	16-ArOPM	Total	16-$ArOH_N$	16-$ArBr_N$	16-$ArOPM_N$
0	30.42	23.50	0	69.3	29.4	0	98.7	70.2	29.8	0
10	33.75	25.43	0.44	75.0	31.0	0.54	106.6	70.4	29.2	0.51
30	32.89	24.11	1.31	71.4	28.7	1.56	101.6	70.2	28.3	1.54
50	29.33	20.73	1.53	72.2	28.0	2.07	102.3	70.6	27.4	2.03
100	32.50	21.32	3.43	70.5	25.4	4.09	99.9	70.6	25.4	4.09
150	33.31	20.24	4.63	72.3	24.1	5.52	101.9	71.0	23.7	5.41
200	34.62	19.18	5.34	75.1	22.9	6.36	104.3	72.0	21.9	6.10

Table S3. HPLC average peak areas, observed and normalized (subscript N) product yields for dediazoniation reaction of 16-ArN_2^+ in solutions of 0.1 M CTAB/0.05 M KBr in the present of 0 – 200 mM anisyl alcohol at 25ºC. [HBr] = 1 mM.

[AniOH]	Peak Areas (10^2 mAU·s)			Observed Yields (%)				Normalized Yields (%)		
(mM)	16-ArOH	16-ArBr	16-ArOAni	16-ArOH	16-ArBr	16-ArOAni	Total	16-$ArOH_N$	16-$ArBr_N$	16-$ArOAni_N$
0	30.42	23.50	0	69.3	29.4	0	98.7	70.2	29.8	0
10	33.24	24.65	0.40	72.1	29.4	0.32	101.8	70.9	28.9	0.32
30	33.26	24.36	0.80	72.2	29.0	0.64	101.8	70.9	28.5	0.63
50	30.18	19.75	1.21	74.3	26.7	1.09	102.1	72.8	26.2	1.07
100	33.58	22.58	2.62	72.9	26.9	2.08	101.8	71.6	26.4	2.04
150	35.06	20.30	3.44	76.1	24.2	2.73	103.0	73.9	23.5	2.65
200	35.73	19.11	4.30	77.5	22.8	3.41	103.7	74.8	22.0	3.30

Section IV: Interfacial molarities

Table S4 Estimated values of interfacial molarities of Br_m, H_2O_m, and $CinOH_m$, in aqueous solutions of 0.1 M CTAB/0.05 M KBr containing 0 – 200 mM cinnamyl alcohol at 25ºC. [HBr] = 1 mM.[a]

[CinOH] (mM)	Br_m[a] (M)	H_2O_m[b] (M)	$CinOH_m$[c] (M)
0	1.78	43.63	0.00
10	1.64	41.86	2.86
30	1.36	40.68	6.15
50	1.31	40.03	7.16
70	1.14	40.18	8.22
100	0.79	36.98	13.71
150	0.68	36.06	15.17
200	0.58	33.70	17.92

a. Br_m = (%16-$ArBr_N$ – 10.38)/11.04

b. $H_2O_m = S_w^{Br}$ x Br_m (%16-$ArOH_N$)/(%16-$ArBr_N$)

c. $CinOH_m = H_2O_m$ x (%16-$ArOCin_N$)/[S_w^{CinOH} x (%16-$ArOH_N$)]; S_w^{CinOH} $\approx$1 according to ref[1].

Table S5 Estimated values of interfacial molarities of Br_m, H_2O_m, and $PEOH_m$, in aqueous solutions of 0.1 M CTAB/0.05 M KBr containing 0 – 200 mM phenylethyl alcohol at 25ºC. [HBr] = 1 mM.[a]

[PEOH] (mM)	Br_m[a] (M)	H_2O_m[b] (M)	$PEOH_m$[c] (M)
0	1.78	43.63	0.00
10	1.78	43.10	0.56
30	1.72	43.08	1.01
50	1.59	43.47	1.65
100	1.48	43.56	2.39
150	1.06	45.46	3.50
200	0.98	45.19	4.32

a. Br_m = (%16-$ArBr_N$ – 10.38)/11.04

b. $H_2O_m = S_w^{Br}$ x Br_m (%16-$ArOH_N$)/(%16-$ArBr_N$)

c. $PEOH_m = H_2O_m$ x (%16-$ArOPE_N$)/[S_w^{PEOH} x (%16-$ArOH_N$)]; S_w^{PEOH} $\approx$1 according to ref[1].

Table S6. Estimated values of interfacial molarities of Br_m, H_2O_m, and $PMOH_m$, in aqueous solutions of 0.1 M CTAB/0.05 M KBr containing 0 – 200 mM phenyl methyl alcohol at 25ºC. [HBr] = 1 mM.[a]

[PMOH] (mM)	Br_m^a (M)	$H_2O_m^b$ (M)	$PMOH_m^c$ (M)
0	1.78	43.63	0.00
10	1.74	43.66	0.31
30	1.67	43.57	0.95
50	1.59	43.84	1.27
100	1.42	43.81	2.54
150	1.29	44.00	3.35
200	1.15	44.58	3.77

a. Br_m = (%16-ArBr$_N$ – 10.38)/11.04

b. $H_2O_m = S_w^{Br}$ x Br_m (%16-ArOH$_N$)/(%16-ArBr$_N$)

c. $PMOH_m = H_2O_m$ x (%16-ArOPM$_N$)/[S_w^{PMOH} x (%16-ArOH$_N$)]; S_w^{PMOH} ≈1 according to ref[1].

Table S7 Estimated values of interfacial molarities of Br_m, H_2O_m, and $AniOH_m$, in aqueous solutions of 0.1 M CTAB/0.05 M KBr containing 0 – 200 mM anisyl alcohol at 25ºC. [HBr] = 1 mM.[a]

[AniOH] (mM)	Br_m^a (M)	$H_2O_m^b$ (M)	$AniOH_m^c$ (M)
0	1.78	43.63	0.00
10	1.72	43.88	0.20
30	1.69	43.97	0.39
50	1.48	45.22	0.66
100	1.51	44.41	1.28
150	1.27	45.81	1.65
200	1.15	46.28	2.05

a. Br_m = (%16-ArBr$_N$ – 10.38)/11.04

b. $H_2O_m = S_w^{Br}$ x Br_m (%16-ArOH$_N$)/(%16-ArBr$_N$)

c. $AniOH_m = H_2O_m$ x (%16-ArOAni$_N$)/[S_w^{AniOH} x (%16-ArOH$_N$)]; S_w^{AniOH} ≈1 according to ref[1].

Section V: NMR results for AniOH

Figure S4. ^{1}H NMR spectra of (A) AniOH & (B) 0.1 M CTAB in 0.1M CTAB/0.05M KBr/AniOH system (C) 80 mM AniOH in D_2O.

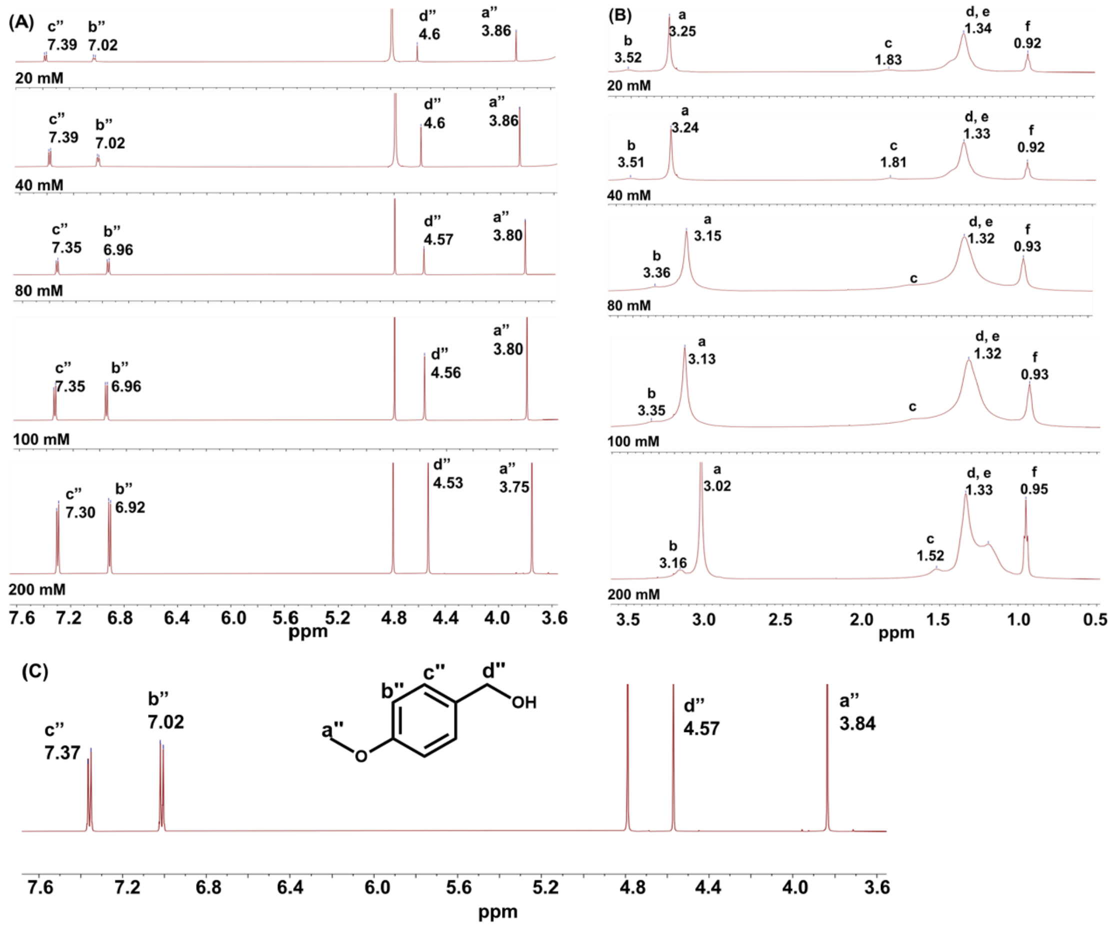

Figure S5. 2D NOESY spectrum of 0.1M CTAB/0.05M KBr/80 mM AniOH in D_2O.

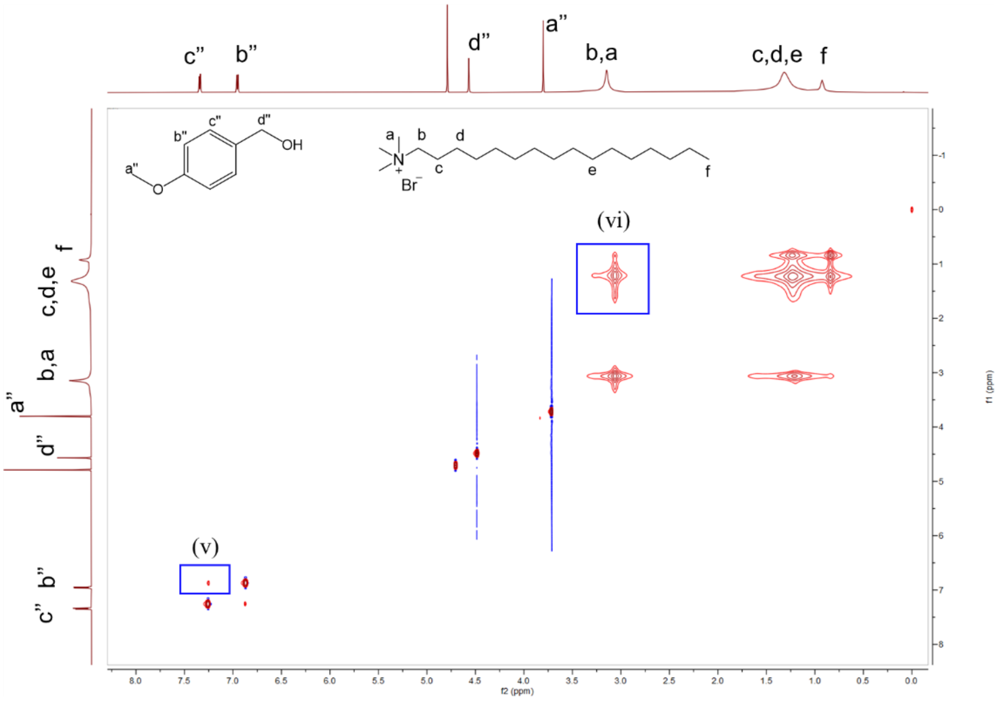

Reference

1. J. Yao, L. S. Romsted, Langmuir, 2000, **16**, 8771–8779.
